# The effects of dietary fiber on common complications in critically ill patients; with a special focus on viral infections; a systematic reveiw

**DOI:** 10.1002/iid3.613

**Published:** 2022-04-19

**Authors:** Azadeh Hajipour, Maryam Afsharfar, Mona Jonoush, Mina Ahmadzadeh, Maryam Gholamalizadeh, Naeemeh Hassanpour Ardekanizadeh, Saeid Doaei, Fatemeh Mohammadi‐Nasrabadi

**Affiliations:** ^1^ Department of Nutrition, School of Health Qazvin University of Medical Sciences Qazvin Iran; ^2^ Department of Nutrition, School of Medicine Zahedan University of Medical Sciences Zahedan Iran; ^3^ Department of Nutrition, School of Medicine Mashhad University of Medical Sciences Mashhad Iran; ^4^ Department of Clinical Nutrition and Dietetics, Faculty of Nutrition and Food Technology, National Nutrition and Food Technology Research Institute Shahid Beheshti University of Medical Sciences Tehran Iran; ^5^ School of Medicine, Cancer Research Center Shahid Beheshti University of Medical Sciences Tehran Iran; ^6^ Reproductive Health Research Center, Department of Obstetrics and Gynecology, School of Medicine, Al‐Zahra Hospital Guilan University of Medical Sciences Rasht Iran; ^7^ Department of Food and Nutrition Policy and Planning Research, Faculty of Nutrition Sciences and Food Technology, National Nutrition and Food Technology Research Institute Shahid Beheshti University of Medical Sciences Tehran Iran

**Keywords:** critically ill patient, dietary fiber, enteral nutrition, viral infection

## Abstract

**Background:**

Viral infections are mostly highly contagious and may cause widespread health problems. Some studies reported that the dietary fiber (DF) may be effective in reducing the complications of viral infections in intensive care unit (ICU) patients. The present review study aimed to investigate the effect of DF on common complications in critically ill patients with viral infections.

**Methods:**

A literature review was conducted for the published papers in English from January 2001 to July 2021 using related keywords. Studies with clinical trial or case‐control design described the effects of fiber intake on the complications of viral infections in patients admitted to the ICU were collected.

**Results:**

DF may reduce the mortality rate of viral infections through modulating inflammatory processes. A higher intake of DF intake may improve hyperglycemia and impaired glucose tolerance in patients with viral infections. A high‐fiber formula in enteral nutrition was reported to reduce the risk of diarrhea in patients with viral infections.

**Conclusion:**

DF may reduce the complications of viral infections such as inflammation, diarrhea, hyperglycemia, and mortality in critically ill patients. Future longitudinal studies on the amount and type of DF are warranted.

## Graphical abstract

Dietary fiber (DF) reduced the risk of chronic diseases through suppressing inflammatory processes. Moreover, increasing DF intake may decrease hyperglycemia and impaired glucose tolerance in patients with viral infections. High‐fiber formula in entral nutrition was reported to reduce the risk of diarrhea in patients with viral infections.

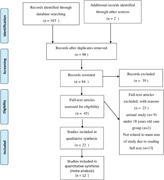

## 1 INTRODUCTION

Viral infections are mostly highly contagious and may cause widespread health problems around the world.^1^ A recent outbreak of unexplained pneumonia in Wuhan, China, led to identifying a new type of coronavirus called severe acute respiratory syndrome coronavirus 2 (SARS‐COV‐2) that causes coronavirus disease 2019 (COVID‐19). This viral infection may be asymptomatic or include various clinical symptoms such as fever, headache, gastrointestinal disorders, dry cough, and shortness of breath. Also, it may have more severe complications such as pulmonary edema, and multiple organ failure that cause patients to require intensive care and may lead to death in severe cases.^2^, ^3^, ^4^

People with viral infections may be hospitalized in the intensive care unit  (ICU) for several days, which may lead to different types of malnutrition, inflammation, acute respiratory distress syndrome, respiratory failure, central or peripheral nervous system manifestations and diarrhea.^5^, ^6^ In hospitalized patients, the prevalence of malnutrition was estimated to be 30%–50% and may cause a higher risk of mortality and morbidity and rehospitalization of the patients.^7^ A significant positive relationship was reported between survival and recovery from the disease with the improvement of the nutritional status of the patients.^8^ Patients admitted to ICU who are unable to consume adequate food for 2 days or more need artificial nutritional support.^9^ If patients' gastrointestinal tract is functioning normally, EN will be considered as the first option to provide nutritional requirements.^7^, ^10^

Some studies reported that the composition of formulas in intenEN may be effective in reducing the complications of acute conditions in ICU patients.^11^, ^12^ The fiber in the formulas used in EN may improve gastrointestinal function by improving the status of gut microorganisms^11^ and reducing the adverse effects of antibiotics on the intestinal microbiota.^13^ One study on high‐fiber diets has demonstrated that the high level of short‐chain fatty acids (SCFAs) in the intestine was associated with reduced lung damage caused by viral infection.^14^ A review study on the effect of intestinal flora against the complications of COVID‐19 indicated that the immune cells produced through induction by a variety of antigens may move in the gut‐lungs axis through the lymphatic system leading to an improved immune response against infections. Improvement of gastrointestinal flora by receiving DF may prevent severe inflammatory reactions in patients with respiratory infection through maintaining the function of the immune system at the desired level.^15^ Moreover, the fiber content of EN in critically ill patients has been effective in reducing the mortality rate and length of stay (LOS) in hospitals.^16^ However, no comprehensive review has been performed regarding the necessity and the proper type and amount of dietary fibers (DF) for the control of complications of viral infections in critically ill patients. Therefore, the present review study aimed to investigate the effect of DF on complications of viral infections in critically ill patients.

## 2 METHODS

### 2.1 Search strategy

A literature search was conducted for the published papers in English from January 2001 to July 2021, using the PubMed, Web of Science, and Scopus databases. The keywords included “fiber” OR “fibers” OR “dietary fiber” OR “dietary fibers” OR “fibre” OR “fibres” OR “dietary fibre” OR “dietary fibres” AND “viral infection” OR “viral infections” OR “viral disease” OR “viral diseases” OR “virus disease” OR “virus infection” OR “covid 19” OR “COVID‐19” OR “SARS‐COV‐2” OR “SARS‐CoV‐2 infection” OR “SARS‐CoV‐2 infections” AND “tube feeding” OR “enteral feeding” OR “enteral nutrition” OR “nutritional support” OR “critical illnesses” OR “critically ill” OR “critical care” OR “intensive care.”

### 2.2 Study selection

The inclusion criteria were applied to collect the papers included studies with clinical trial or case‐control design, described the effects of fiber intake on common complications of viral infections, and performed on patients admitted to the ICU. The animal studies, studies on patients under 18 years of age, and studies that discussed diet generally (not fibers in particular) were excluded.

### 2.3 Data extraction

Data was collectedfrom the studies included general characteristics of the study (the first author, year of publication, study design, and duration of study), aspects of the study population (age, gender, sample size, and disease), experimental intervention (type and amount of DF), and the main outcomes of the studies.

## 3 RESULTS

**Figure 1:**
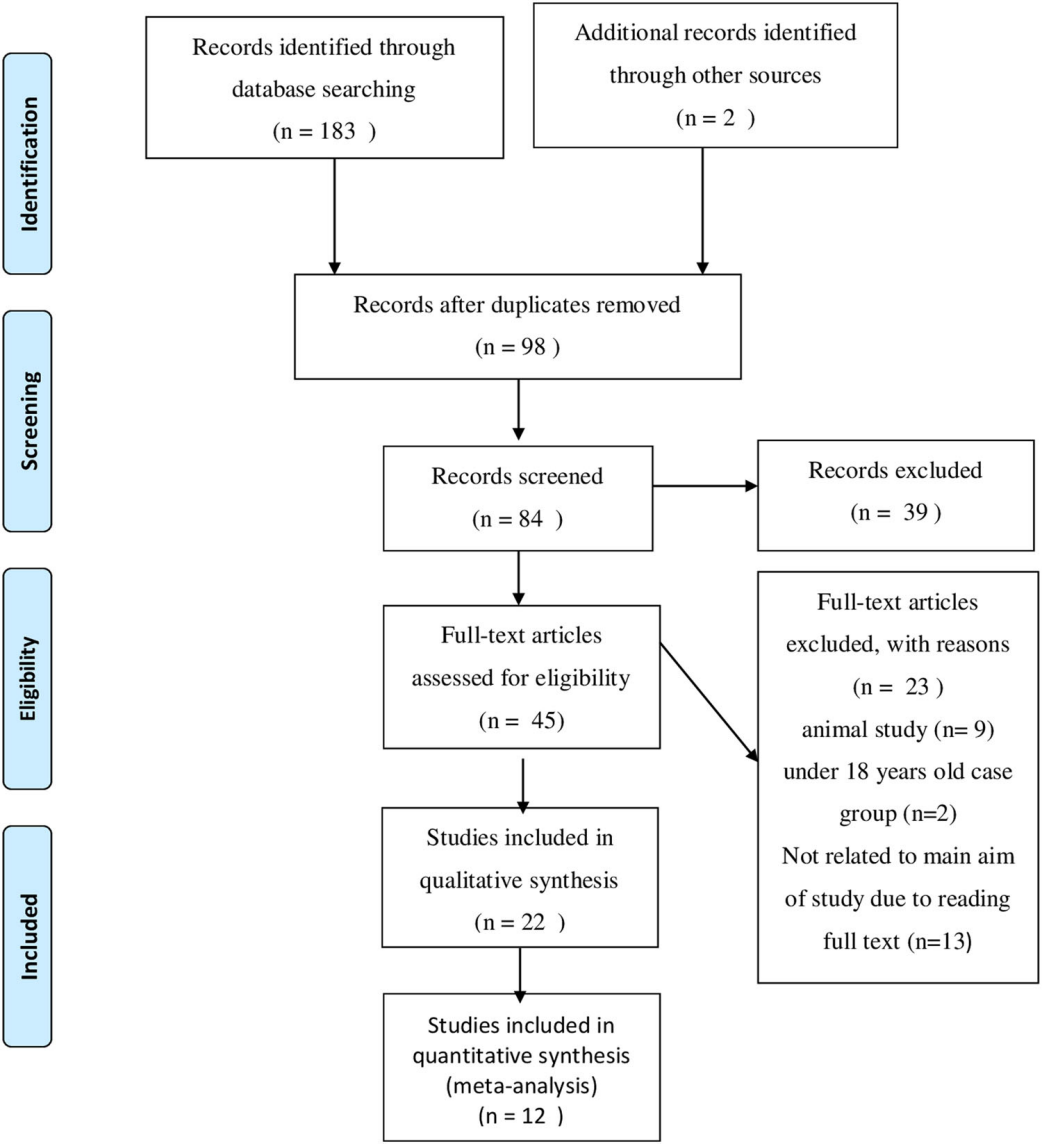
Flow chart of the literature search strategy and syudy selection

We performed a literature search in the PubMed, WOS, and Scopus databases, and 183 articles were initially identified. According to the inclusion and exclusion criteria, 170 duplicate and irrelevant articles were excluded by reading titles and abstracts (Figure 1). After reading the full‐text, 12 studies were finally included in the present review. The results identified that diarrhea, hyperglycemia, acute kidney inflammation (AKI), inflammation, and higher mortality are the most common side effects of viral infections that may be affected by fiber intake. In total, three studies on the effect of fiber on inflammation and lung function, five studies on the effect of fiber on metabolic function and mortality, and four studies on the effect of fiber on diarrhea were included in the systematic review. The detailed characteristics of the studies included in this article are presented in Table 1.

**Table 1:** A summery of relevant studies regarding the effects of dietary fiber on common complications of viral infections

Complications	Authors	Study population	Study design	Main outcome
Lung function and inflammation	Berthon et al.^17^	137 case case and 65 controls	Cross‐sectional	Lower fiber consumption related to lower lung function
				(odds ratio 1.04 [95% confidence interval: 1.01–1.07], *p* = .014)
	McLoughlin et al.^18^	17 patients with stable asthma in 3 × 7 day trial of consuming soluble fibre, soluble fibre + probiotic, or placebo	RCT	Improvements in asthma control, lung function (median [IQR] −0.35 (−0.5, −0.13), *p* = .006)
	Halnes et al.^19^	29 subjects with stable asthma (*n* = 17 case in soluble fiber meal group, *n* = 12 control in simple carbohydrate meal group)	Case‐control	Improvements in inflammation biomarkers (*p* = .02)
The effects of dietary fiber on metabolic disorders and Mortality	Chen et al.^20^	117 patients with DM2 in three treatment group (*n* = 37 Control, *n* = 40 Group 2, and *n* = 40 Group 3)	Randomized, double‐blind trial	Improvention in the levels of fasting blood glucose, insulin resistant, C‐peptide, insulin resistance index (*p* < .05)
	Buil‐Cosiales et al.^21^	7216 elderly adults at high CVD risk	Cohort	Reduction in total mortality
				(HR: 0.59; 95% CI: 0.44, 0.78).
	Katagiri et al.^22^	42,754 men and 50,170 women	Cohort	Inversely association with all‐cause mortality.
				(95% CI: 0.72, 0.82; *p*‐trend <.0001) in men and 0.82 (95% CI: 0.76, 0.89; *p*‐trend <.0001) in women.
	Park et al.^23^	219,123 men and 168,999 women	Cohort	Reduction in mortality from infections (95% CI: 0.73–0.82; *p* for trend, <.001) in men and 0.78 (95% CI: 0.73–0.85; *p* for trend, <.001) in women.
	Salmean et al.^24^	13 CKD patients in two phase trial (phase 1 = 14 days with 1.6gr/day fiber, and phase 2 = 28 days with 23gr/day fiber).	Single‐blind crossover study	Reducing BUN, serum creatinine levels and improving eGFR.
				10.6% decrease in mean BUN concentration (13.8 ± 2.0 to 12.1 ± 1.8 mmol/L or 38.5 ± 5.6 to 34.0 ± 5.1 mg/dl; *p* < .05).
				Serum creatinine level decreased from a baseline value of 216 ± 26 to 201 ± 23 mmol/L (2.44 ± 0.30 to 2.27 ± 0.26 mg/dl; *p* < .05) after 2 weeks
The effects of dietary fiber on Diarrhea	Zhao et al.^25^	120 intensive care patients with gastric cancer in 3 groups (40 subjects in each group): control, fiber intake, and fiber + probiotic‐intake, **with tube feeding**	A prospective randomized and controlled trial	Reduction in incidence of diarrhea (*p* = .003)
	Jakobsen et al.^26^	51 patients **with tube feeding** in ICU(test: n = 26 dietary fiber enriched **enteral formula**, control: n = 25 isocaloric non‐enriched formula)	RCT	Reduction in incidence of diarrhea in patients receiving the test formula compared with the control group (19% vs. 48%, *p* = .034).
	Fu et al.^27^	129 critically ill patients **with tube feeding in ICU**	Cohort	No association with diarrhea.
				(15% in high fiber vs. 13% in no fiber, *p* = .94)
	Yagmurdur et al.^28^	120 patients **with tube feeding in ICU**	Case‐control	Higher volume ratio values and lower incidence of diarrhea (*p* < .001).
		Control (*n* = 60) case (*n* = 60)		

Abbreviations: BUN, blood urea nitrogen; CI, confidence interval; CKD, chronic kidney damage; DM2, diabetes mellitus type 2; eGFR, estimated glomerular filtration rate; HR, hazard ratio; ICU, intensive care unit; RCT, randomized control trial.

## 4 DISCUSSION

In this review study, it was found that DF may affect the control of complications such as diarrhea, inflammation, mortality of hospitalized patients, control of blood sugar, and blood factors that affect kidney function. Because these factors are a common complication of patients with viral infections admitted to the ICU, it is possible to take advantage of the following benefits of fiber in the intestinal nutrition of these patients.

### 4.1 DF and inflammation

A negative association was reported between DF and systemic inflammation.^29^ DF decreases the risk of chronic diseases through modulating inflammatory processes.^30^ It was reported that improving the gastrointestinal flora through receiving DF may prevent severe inflammatory responses in patients with viral infections by maintaining the function of the immune system at the optimum level.^15^ So that increased fiber intake was significantly associated with lower odds of having an elevated C‐reactive protein concentration and a positive effect on the interleukin‐6 reduction when compared with the lowest intake.^31^, ^32^, ^33^

The SCFAs including butyrate, acetate, propionate, which are produced through bacterial fermentation of DF in the colon,^34^ have independent beneficial roles in gut defense against viruses, production of secretory immunoglobulin A, and anti‐inflammatory responses.^35^ SCFAs also may inhibit nuclear factor kappa light chain enhancer of activated B cells (NFкB) activity through activating of G protein‐coupled receptor (GPR) 41 and 43, and exert some anti‐inflammatory effects through reduced neutrophil migration, decreased production of proinflammatory cytokines, and decreased adhesion molecule expression.^18^ Furthermore, butyrate may increase the peroxisome proliferator‐activated receptor‐α which then decreases nuclear factor kappa B activity. Moreover, SCFAs have epigenetic effects by inhibition of histone deacetylase enzyme. Berthon et al.^17^ reported that a lower fiber and potassium diet in 137 stable asthmatic patients was related to increased airway inflammation and decreased lung function.

**Figure 2:**
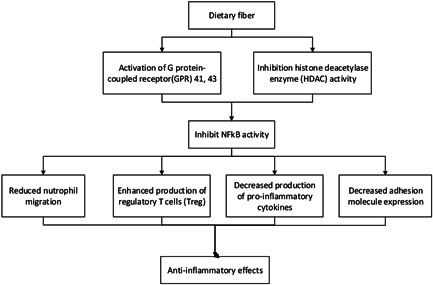
The possible effects of dietary fiber on inflammation in critically ill patients

DF water solubility has an important effect on its availability for the gut microbiota and leads to a higher fermentation of dietary soluble fiber than insoluble fiber.^36^ The soluble fibers undergo degeneration by anaerobic bacteria present within the colon, resulting in the assembly of many metabolic by‐products, like SCFAs and lactate.^37^ The SCFAs produced by *Bifidobacteria* and *Lactobacilli* lower luminal pH, preferentially stimulating the expansion of those classically “health‐promoting” bacteria and inhibiting the proliferation of “harmful” bacteria, including enteric pathogen *Escherichia coli*.^30^ The soluble fibers may cause more biodiversity of the gut microbiome than insoluble fiber, which is associated with increased protection against inflammation.^38^ Interestingly, a study on asthma patients found that taking 3.5 g of inulin with probiotic yogurt resulted in a reduction in inflammation and improved lung function compared to the controls after 4 h. Also, this study supported that intervention increased GPR41 and GPR43 expression, suggestive that mechanisms related to SCFA‐mediated free fatty acid receptor activation could also be related to anti‐inflammatory action in the asthma patients' airway.^19^ A randomized cross‐over trial on the soluble fiber supplementation effects on airway inflammation revealed that inulin supplementation decreased eosinophilic‐induced airway inflammation.^18^ It is plausible that increasing the consumption of rich sources of soluble fibers such as fruits, vegetables, and whole grains regulates the innate and adaptive immune systems to fight against viral infection^39^ (Figure 2).

On the other hand, consuming insoluble fiber may regulate the immune system in several ways, including mechanical autophagy that helps remove infectious agents^40^ and may alleviate these effects through binding of produced SCFAs to the GPRs.^30^ Totally, recent studies suggest that a higher intake of DF may be considered in the reduction of inflammation in patients with viral infections (Table 1).

### 4.2 DF and glycemic control

Patients with viral infections are at higher risk of hyperglycemia which may lead to glucose toxicity and tissue damage.^41^ Patients with COVID‐19 who have hyperglycemia were reported to have higher hospital LOS and higher mortality.^42^ In patients with COVID‐19, insulin resistance has been attributed to several mechanisms such as islet cell infection by direct binding of coronavirus to angiotensin‐converting enzyme 2 on β‐cells and the effect of elevated cytokine levels on impairments of β‐cells.^43^ Furthermore, viral infections may cause insulin resistance because of the downregulation of insulin receptor expression in skeletal muscle due to virus‐induced interferon gamma. Muscle insulin resistance results in compensatory hyperinsulinemia to keep blood glucose in a normal range.^44^ According to another hypothesis, coronavirus may infect adipocytes and induce adipose tissue dysfunction resulting in lower levels of adiponectin. Finally, decreased levels of adiponectin lead to insulin resistance and hyperglycemia.^42^

**Figure 3:**
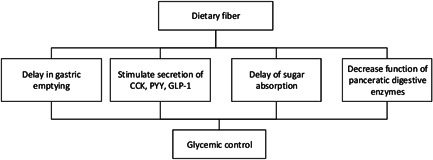
The possible effects of dietary fiber on glycemic control in critically ill patients

Soluble fibers are associated with an improvement in the glucose and lipid profile through delay in gastric emptying (GE). In addition, the production of SCFAs in the colon following DF fermentation may reduce the absorption of carbohydrates.^45^ These fermented products stimulate the secretion of gut‐derived hormones such as glucagon‐like peptide1, cholecystokinin, and peptide YY (PYY), which reduce GE.^46^ Thus, by delayed GE, the level of available carbohydrates for digestion and absorption may decrease significantly.^47^ Another potential mechanism of DF role in glycemic control included a decreased function of pancreatic digestive enzymes, especially alpha‐amylase, which in turn reduces glucose production.^47^ Chen et al.^20^ assessed the effects of soluble DF on glycemic control in patients with diabetes mellitus type 2. Patients were randomly assigned to receive DF (10 or 20 g/day), or control groups (0 g/day). Following 30 days of the intervention, the levels of insulin secretion, 2‐h blood glucose, lipoprotein (a), the insulin resistance index, and C‐peptide were significantly improved in the soluble DF groups. In general, according to the studies, increasing DF intake especially soluble DF may improves hyperglycemia and impaired glucose tolerance in patients with viral infections (Figure 3).

### 4.3 DF and diarrhea

Diarrhea may be one of the first signs of viral infections.^48^ A recent study in China reported that about 10% of the patients with COVID‐19 admitted to the hospital had diarrhea, and 24% of them had nausea and vomiting.^49^ In addition, between 3% and 78% of critically ill patients experience diarrhea.^50^ However, there is concern that fiber can worsen diarrhea and cause bloating during critical illnesses, but some studies reported contradictory results. A prospective ICU‐based cohort study on 129 critically ill patients indicated that higher fiber intake is related to higher levels of SCFA produced by bacteria, but not associated with adverse gastrointestinal events, including diarrhea.^27^ Another study on 120 intensive care patients admitted for gastric cancer to compare the effect of enteral feeding containing fiber and probiotics compared to a fiber‐free diet on diarrhea after 7 consecutive days reported that the patients who received fiber and probiotics experienced improved intestinal movements and less diarrhea. Furthermore, the addition of fiber to EN formulas effectively prevented diarrhea compared with fiber‐free EN formulas.^25^ DF may influence gastrointestinal transport through its effect on the neuroendocrine system. The production of SCFAs after fiber fermentation alters the secretion of hormones such as PYY, which is accounted to stimulate the absorption of electrolytes and water and control the ileal brake which is defined as a distal to a proximal feedback mechanism to control the transit of a meal through the gastrointestinal tract.^51^ Also, the SCFAs are rapidly absorbed by the intestinal cells, arousing fluid absorption through the noncyclic‐AMP activation of sodium absorption.^52^ Other proposed mechanisms of the role of DF in preventing diarrhea are improving gut barrier function and increasing epithelial cell turnover or regeneration.^53^

The regulation of the immune system may be impaired during critical viral infections due to gut dysbiosis. Thus, nutritional therapy should focus on promoting a healthy gut microbiome.^54^ Early EN including soluble fiber and probiotics may help sustain the growth of commensal organisms and maintain gut barrier defense. Recent guidelines of the American Society for Parenteral and Enteral Nutrition suggest that soluble fibers may be recommended to all patients in the ICU.^35^

A study compared the results of a fiber‐enriched formula with an isocaloric nonenriched formula in ICU patients with an indication of enteral feeding (TF) and reported that an TF formula enriched with fiber might positively affect GI tolerance.^55^ Furthermore, the rate of diarrhea was lower in the patients receiving the fiber‐enriched formula compared with the control group (19% vs. 48%, *p* = .034).^26^

**Figure 4:**
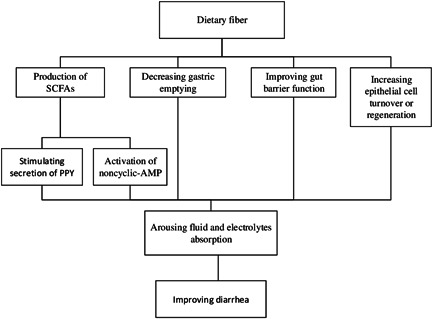
The possible effects of dietary fiber on Diarrhea in critically ill patients

These findings suggest that EN in ICU patients with viral infections may be started with fiber‐enriched formulas rather than fiber‐free formulas to avoid frequent feeding interruptions that cause protein‐energy malnutrition in ICU patients^28^ (Figure 4).

### 4.4 DF and mortality

The higher levels of serum inflammatory biomarkers in critical patients with viral infections are associated with higher mortality and the possible effect of DF on reducing mortality may be due to its role in reducing inflammation.^56^ In a recent cohort study, the consumption of DF intake was associated with a lower mortality from infections and lung diseases after 9 years of follow‐up. An increase in DF as 10 g/day was associated with a lower risk of infectious diseases and respiratory diseases in men (relative risk [RR] = 0.66, confidence interval [CI]: 0.52–0.84 and [RR] = 0.82, CI: 0.74–0.93, respectively) and women (RR = 0.61, CI: 0.44–0.85 and RR = 0.66, CI: 0.56–0.78, respectively). Another cohort study in Japan on 92,924 participants after 15 years follow‐up with 19,400 deaths reported that DF intake was related to lower all‐cause and cause‐specific mortality. The highest fiber consumption was reported to be associated with 23% and 18% lower mortality compared with the lowest fiber consumption in men and women, respectively.^22^ Furthermore, another cohort study of elderly adults over 5.9 years of mean follow‐up indicated that a higher intake of fiber was related to a reduced rate of total mortality.^21^

**Figure 5:**
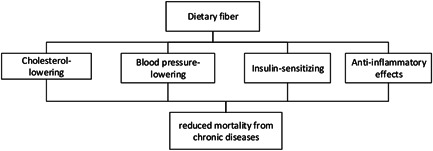
The possible effects of dietary fiber on mortality rate in critically ill patients

Acute kidney injury (AKI) is a common complication of viral infections in severe conditions^57^ and may increase the risk of death up to 9 times,^58^ especially when AKI resultes in dialysis.^59^ A prospective cohort study of 1603 hospitalized patients to assess acute and chronic kidney damage (CKD) during the COVID‐19 epidemic serum creatinine increased in 21% of patients, and patients with elevated serum creatinine had a higher mortality rate than those with normal creatinine.^60^ A 6‐week single‐blind cross‐over study on the effect of fiber on blood urea nitrogen and serum creatinine concentrations in patients with CKD reported that increasing fiber reduces serum creatinine levels and improves estimated glomerular filtration rate (eGFR).^24^ The ability of DF in improving blood cholesterol level, blood pressure, and insulin‐sensitivity may be associated with reduced mortality.^22^ Overall, studies have shown that high DF intake may reduce mortality in patients with viral infections by improving their severe complications, especially renal failure (Figure 5).

## 5 CONCLUSION

The results of studies on the effect of fiber on complications of viral infections have shown that the consumption of soluble and insoluble fiber in one's diet may reduce the complications of the disease by various mechanisms such as improving inflammation, diarrhea, and hyperglycemia. It may also reduce the mortality rate in critically ill patients. Future longitudinal studies on the appropriate amount and type of fiber for recommendation in ICU patients with viral infections are warranted.

## CONFLICTS OF INTEREST

The authors declare no conflict of interest.

## AUTHOR CONTRIBUTIONS

**Azadeh Hajipour, Maryam Gholamalizadeh, Maryam Afsharfar, Mona Jonoush, Mina Ahmadzadeh, Naeemeh Hassanpour Ardekanizadeh and Saeid Doaei**: designed the study, involved in the data collection, analysis, and drafting of the manuscript. **Saeid Doaei and Fatemeh Mohammadi‐Nasrabadi**: were involved in the design of the study, analysis of the data, and critically reviewed the manuscript. All authors read and approved the final manuscript.

## Data Availability

The data of this article will be made available by the authors, if they are requested, to any qualified researcher.
